# Community practices of face mask use and associated knowledge and attitude in a Malaysian town during the COVID-19 pandemic

**DOI:** 10.4314/gmj.v57i2.4

**Published:** 2023-06

**Authors:** Husna A. Jalaluddin, Ismail A. S. Burud, Regina P. C. Yew, Xiao Thoong Loh, Quintina G. J. Tan, Navinea Nathan, Aqil M. Daher

**Affiliations:** 1 School of Medicine, International Medical University, Clinical Campus, Seremban, Malaysia; 2 Department of Surgery, International Medical University, Clinical Campus, Seremban, Malaysia; 3 Department of Community Medicine, International Medical University, Kuala Lumpur, Malaysia

**Keywords:** Attitudes, Community, COVID-19, Face masks, Knowledge, Practice

## Abstract

**Objectives:**

Among the vital measures to effectively curb the incidence of COVID-19 is the use of face masks. Little is known about what people understand and how they perceive and use face masks. This study aimed to determine the community's knowledge, attitude, and practice on the correct use of face masks.

**Design:**

A cross-sectional study.

**Setting:**

The study was carried out in Seremban 2, Malaysia using a self-administered questionnaire adapted from validated questionnaires of two previous studies.

**Participants:**

Through opportunistic sampling, three hundred and ninety-two literate adults (above 18) residing in Seremban 2, Malaysia, participated in this study.

**Main outcome measure:**

Knowledge, attitude, and practice scores were assessed among the participants.

**Results:**

Three hundred seventy (94.4%) respondents demonstrated satisfactory knowledge. A satisfactory attitude score was achieved by 349 (89%) respondents, while 281 (71.7%) achieved a satisfactory practice score. Better knowledge was significantly associated with college or university education (p=0.028). Female gender (p=0.011) and college or university education (p=0.043) were significantly associated with better practice (p<0.05). Significant but weak to fair correlations between knowledge, attitude and practice were observed.

**Conclusion:**

Overall, there was satisfactory knowledge, attitude, and practice of face mask use among the Seremban 2 adult population in Malaysia. However, future public health education targeted toward the use of face masks requires more emphasis on proper usage and disposal to translate good knowledge into a good attitude and practice of face mask use to ensure the effectiveness in curbing the spread of infection.

**Funding:**

None declared

## Introduction

Since its declaration as a pandemic in March 2020, severe acute respiratory syndrome coronavirus 2 (SARS-CoV-2), also known as COVID-19, has affected almost every part of the world and jeopardised various aspects of normal life.^1^ It is well-documented that COVID-19 is transmissible through respiratory droplets and may even be airborne. As such, to prevent transmission, the World Health Organization (WHO) recommendations are to practice frequent hand hygiene, physical distancing from others when possible, have respiratory etiquette, and use face masks.^2^ The use of face masks has been proven to reduce the transmission of respiratory droplets, based on studies by Leung et al.^3^ and Wang et al.^4^ conducted in Hong Kong and Beijing, respectively.

N95 masks are more effective than surgical face masks in preventing respiratory infection among healthcare workers based on two separate randomised clinical trials by Macintyre et al.^5^,^6^ However, there is some controversy regarding the use of cloth face masks as their efficacy is not very well-proven as concluded by Mahase^7^ and Macintyre et al.^8^

The WHO has since released guidelines on how and when to wear face masks.^9^ Medical masks, also known as surgical masks, are recommended for all healthcare workers in clinical settings, those who are unwell, and those caring for suspected or confirmed cases of COVID-19.^9^

Medical masks are also recommended for high-risk groups, such as people aged 60 or over and those of any age with underlying comorbidities.^9^ Non-medical fabric masks are advised for use by the public when physical distancing cannot be maintained.^9^ Respirator masks (FFP2, FFP3, N95, N99) should be reserved for healthcare workers performing aerosol-generating procedures.^5^,^9^ According to the Centers for Disease Control and Prevention of the United States (CDC), the use of face masks should be supplementary to other preventive methods such as handwashing, avoiding close contact, and proper coughing and sneezing etiquette.^10^

Since then, recommendations on face mask use have evolved slightly. The WHO in January 2022 recommended the public use face masks in settings where there is community or cluster transmission of COVID-19, irrespective of vaccination status or history of prior infection, and also when interacting with individuals outside of the household.^11^ In the United States, rules and mandates on face mask use have relaxed significantly since April 2022, allowing face mask use based on preference in counties with lower COVID-19 case numbers.^12^ However, many lessons can be learnt from the handling of the COVID-19 pandemic to be applied to future instances, including the aspect of face mask use.

Increased awareness of influenza-like symptoms and transmission of infection among the Hong Kong community prevented the spread of the H5N1 influenza virus from progressing to a pandemic in 2008, indicating the importance of community awareness in preventing infectious diseases.^13^ A study from Ethiopia by Tadesse et al.^14^ showed less than satisfactory practices in face mask use even among healthcare workers. However, Azlan et al.^15^ and Ahmed et al.^16^ showed good knowledge, attitude, and practices (KAP) towards face mask use during the COVID-19 pandemic, although this was opposed by Alam et al.^17^ Other studies from Ho^18^ and Goni et al.^19^ have previously drawn a significant but weak correlation between variables, especially attitudes and practices. So far, little is known about the community's KAP towards face masks in Malaysia, specifically after the COVID-19 pandemic. This study aimed to determine the knowledge regarding the use of face masks within the Seremban 2, Malaysia community, and how it influences their attitude and practices.

## Methods

A cross-sectional study was carried out in Seremban 2, a satellite town in Malaysia, for 2.5 months from September to November 2020. The literacy level of adults in Malaysia was 94.97% in 2019, with no separate data for individual states.^20^ Adult respondents 18 years and above, literate, and living near Seremban 2 were included. The sample size was calculated through the Raosoft sample size calculator using the estimated population of 62 000 in Seremban 2 with a confidence level of 95% and a confidence interval of 5.^21^ The estimated sample size was 382. Adjusting for a 10% non-response rate, the targeted participants were 420. Opportunistic sampling was used in this study by handing out questionnaires to the participants in public areas such as malls, clinics, and eateries.

After getting permission from the respective authors, the self-administered questionnaire was adapted and modified from two validated questionnaires created by Ho^18^ and Goni et al.^19^. The questionnaire was initially constructed in English and translated into Malay using a forward-backwards method to ensure linguistic, semantic, and cultural equivalence. The original meaning was not lost during translation. It was piloted on a small sample, and minor errors were modified. The questionnaire consists of four components: demographic details (age, gender, ethnicity, marital status, level of education, and occupation), and KAP on the use of face masks.

To assess the participants' knowledge, they were asked about the correct ways of using a face mask, the purpose of face mask use, and appropriate occasions to use it. Good attitudes were established by questions regarding the perception of using a face mask. In the ‘Practice’ component, participants were asked about the occasions when they personally used face masks and their practice of the correct method of using and disposing of face masks. This evaluates the compliance and uptake of various preventive measures involving face masks. The questionnaire also enquired about their preferred face mask and the use of reusable face masks, respirators and cloth face masks. While using reusable face masks, good practice includes storing them properly before re-using and sanitising them appropriately.

### Scoring

There were 12 questions in the ‘Knowledge’ component answered with ‘Yes’, ‘No’, or ‘I don't know’. A score of 1 was given for each correct response, whereas a response that was incorrect or ‘I don't know’ would be given a score of 0, yielding a minimum score of 0 and a maximum score of 12. Six questions for the ‘Attitude’ component were answered based on a Likert scale ranging from ‘strongly disagree’, ‘disagree’, ‘uncertain’, ‘agree’ and ‘strongly agree’. The ‘Attitude’ component had scores ranging from 5, 4, 3, 2, and 1, with the highest score allocated to the answer supporting good attitudes and vice versa. The aggregated scores ranged from 6 to 30. To assess practice, we provided the options of ‘always’, ‘occasionally’, and ‘never’ for 14 questions. The correct practices should always be followed, not just occasionally or never.

Therefore, the “Practice” component was scored with good practice “Always”, given a score of 1. In contrast, those practices carried out “Occasionally” or “Never” was given a score of 0, a minimum score of 0 and a maximum score of 14. Referring to two previous KAP studies, satisfactory knowledge, attitude, and practice were each defined by a cut-off point of 70% or more, whereas a score of less than 70% was considered unsatisfactory.^22^,^23^

### Data analysis

IBM SPSS statistics software version 24.0 was used to analyse the data. The categorical variables of this study were described with frequency and percentage. Numerical variables were described with mean and standard deviation. Pearson's chi-square test was used to test the association between socio-demographic variables and preference for face masks with the KAP scores. The correlation coefficient was calculated by Spearman's correlation to measure the strength of the association between KAP. A score of 0.0 to 0.09 was defined as having no correlation; 0.1 to 0.3 as a small correlation; 0.3 to 0.5 as a medium correlation; 0.5 to 1.0 as a large correlation.^24^ A p-value of 0.05 or lesser was considered statistically significant.

### Ethical considerations

Verbal and written consent was obtained before the distribution of the questionnaires. The International Medical University (IMU) Joint Committee on Research & Ethics, International Medical University provided approval number 4.8/JCM-202/2020 with project ID number CSc/Sem6(17)2020.

## Results

The response rate was 93.3%, as 392 participants out of 420 returned the completed questionnaire. Therefore, the sample size was 392. The details of the socio-demographic aspects are shown in Table 1.

**Table 1:** Distribution of respondents by socio-demographic characteristics (n=392)

Socio-demographic characteristics		n (%)
**Age**	< 40	223 (56.9)
	> 40	169 (43.1)
**Gender**	Male	126 (32.1)
	Female	266 (67.9)
**Race**	Malay	195 (49.7)
	Non-Malay	197 (50.3)
**Marital Status**	Single/divorced	196 (50.0)
	Married	196 (50.0)
**Education**	Primary/secondary school	102 (26.0)
	College/university	290 (74.0)
**Occupation**	Student/unemployed	104 (26.5)
	Non-medical	261 (66.6)
	Medical	27 (6.9)

Regarding preference of face mask type, 324 (82.7%) participants preferred wearing surgical face masks. Respirators (N95, KN95) and cloth face masks were preferred almost equally, with 84 (21.4%) and 83 (21.2%) participants, respectively. Only 10 (2.6%) had sponge face masks as their preferred choice. Other options of face masks available in the market were only preferred by 4 (1%) participants (data not shown in the tables).

### Knowledge

A majority, 370 (94.4%), of participants had satisfactory knowledge of the proper use of face masks. The mean (SD) of the total knowledge score of the participant was 10.70 (1.3). Participants' knowledge of the use of face masks is summarised in Table 2.

**Table 2:** Responses regarding knowledge of the use of face masks (n=392)

Responses to knowledge questions	n (%)	
	Correct response	Incorrect response
I am aware that the face mask should cover both the mouth and nose.	391 (99.7)	1 (0.3)
The metal strip on the face mask is to fit on the nose.	388 (99.0)	4 (1.0)
The proper side of the face mask is such that the coloured side is facing outwards.	379 (96.7)	13 (3.3)
The different types of face masks available in the market are surgical face masks, cloth face masks, and N95 masks.	351 (89.5)	41 (10.5)
A cloth face mask is not as effective as a regular 2-ply surgical face mask.	170 (43.4)	222 (56.6)
Surgical face masks and N95 masks are not reusable with correct handling in between uses.	173 (44.1)	219 (55.9)
A face mask can protect me from airborne infections like colds and influenza by preventing me from inhaling air droplets containing microorganisms.	370 (94.4)	22 (5.6)
To prevent infection face masks should be used in combination with frequent handwashing with soap and water or with alcohol-based hand sanitiser.	385 (98.2)	7 (1.8)
**Prioritization:**		
Healthcare workers (e.g.: doctors, nurses, etc.)	373 (95.2)	19 (4.8)
Individuals with respiratory infection symptoms (e.g.: coughing, sneezing, and fever)	379 (96.7)	13 (3.3)
Individuals taking care of or being in close contact with someone with a respiratory infection.	371 (94.6)	21 (5.4)
Individuals that are working in close contact jobs (e.g.: barbers etc.) or food handlers	367 (93.6)	25 (6.4)

### Attitude

Most participants, 349 (89%), achieved satisfactory attitude scores towards using face masks. The data collection gave a mean of 25.13 and an SD of 3.4. A striking total number of 377 (96.1%) participants strongly disagreed or disagreed that they would only wear a face mask if it were given for free.

Regarding the belief that face masks are useless in preventing the spread of airborne infection, 230 (58.7%) participants strongly disagreed, and 124 (31.6%) disagreed with the statement. One hundred and twenty (30.6 %) participants agreed or strongly agreed that wearing a face mask makes breathing and speaking difficult. (Table 3).

**Table 3:** Responses regarding attitude on the use of face masks (n=392)

Responses to attitude questions		n (%)
I know the proper steps to wear and dispose of a face mask correctly.	Strongly disagree	41 (10.5)
	Disagree	14 (3.6)
	Uncertain	21 (5.4)
	Agree	157 (40.1)
	Strongly agree	159 (40.6)
I will only wear a face mask if it is given for free.	Strongly disagree	244 (62.2)
	Disagree	133 (33.9)
	Uncertain	10 (2.6)
	Agree	5 (1.3)
	Strongly agree	0 (.0)
I believe that face masks are of no use in preventing the spread of airborne infection.	Strongly agree	7 (1.8)
	Agree	10 (2.6)
	Uncertain	21 (5.4)
	Disagree	124 (31.6)
	Strongly disagree	230 (58.7)
I am shy to wear a face mask in public.	Strongly agree	1 (0.3)
	Agree	2 (0.5)
	Uncertain	4 (1.0)
	Disagree	118 (30.1)
	Strongly disagree	267 (68.1)
Wearing a face mask makes it difficult for me to speak and breathe.	Strongly disagree	88 (22.4)
	Disagree	146 (37.2)
	Uncertain	38 (9.7)
	Agree	109 (27.8)
	Strongly agree	11 (2.8)
If I have an airborne infection, I may infect others if I do not wear face masks.	Strongly disagree	45 (11.5)
	Disagree	13 (3.3)
	Uncertain	33 (8.4)
	Agree	99 (25.3)
	Strongly agree	202 (51.5)

### Practice

The mean (SD) of the total practice is 10.77 (2.38). Among 392 participants, 281 (71.7%) had achieved satisfactory practice scores using face masks. Table 4 shows the respondent's practice on the use of face masks.

**Table 4:** Responses regarding practice on the use of face masks (n=392)

Responses to practice questions	n (%)	
	Never or Occasionally	Always
I wear a face mask in public venues to protect myself against airborne infections.	15 (3.9)	377 (96.2)
I wear a face mask in healthcare facilities to protect myself against airborne infections.	16 (4.1)	376 (95.9)
I wear a face mask if I have symptoms of an airborne infection.	31 (7.9)	361 (92.1)
I wear a face mask when I am in contact with someone who has an airborne infection.	30 (7.7)	362 (92.3)
I remove my face mask when I talk to someone.	365 (93.1)	27 (6.9)
I am not careful regarding the way I keep my face mask and dispose of it.	359 (91.6)	33 (8.4)
I started using face masks only after the COVID-19 pandemic.	30 (7.6)	362 (92.3)
I am particular about using face masks more often after the COVID-19 pandemic.	40 (10.2)	352 (89.8)
**When using face masks:**		
I make sure my face mask covers both the nose and mouth with the folds facing downwards on the outside.	43 (11.0)	349 (89.0)
I ensure it fits properly and there are no gaps or holes on the sides and front.	62 (15.8)	330 (84.2)
I am careful to not touch the front part of the face mask	152 (38.8)	240 (61.2)
I wash my hands for at least 20 seconds whenever I touch the front of the face masks or after removing them	201 (51.2)	191 (48.7)
I dispose of used face masks in a closed bin.	100 (25.5)	292 (74.5)
I re-use my disposable face mask.	351 (89.6)	41 (10.5)

Of 392 participants, only 182 (46.4%) always and occasionally use reusable face masks like cloth face masks and respirators. Among the 182, 131 (72%) had satisfactory practice when re-using face masks, while the other 52 (38%) had poor practice. The mean (SD) of the total practice when re-using reusable face masks is 4.19 (1.2) (data not shown in the tables).

### Association of Demographic Characteristics and KAP Scores

It was noted that participants with college or university education (p=0.028) were significantly associated with achieving satisfactory knowledge scores on the use of face masks. None of the demographic characteristics was found to be statistically significant in association with the attitude of the participants in this study. However, females (p=0.011) and participants with college or university education (p=0.043) were significantly associated with satisfactory practice (p<0.05). The demographic variables are not statistically significant to most KAP scores. (Table 5).

**Table 5:** Association between satisfactory knowledge, attitude, and practice on the use of face masks and the socio-demographic characteristics of the participants

	Knowledge			Attitude			Practice		
	n (%)	p-value	OR (95%CI)	n (%)	p-value	OR (95%CI)	n (%)	p-value	OR (95%CI)
Age:									
**< 40 years old**			1			1			1
**> 40 years old**	164 (97.0)	0.109	2.640 (0.806-8.649)	145 (85.8)	0.140	0.548 (0.247-1.218)	131 (77.5)	0.363	1.300 (0.739-2.285)
Gender:									
**Male**			1			1			1
**Female**	252 (94.7)	0.939	0.964 (0.378-2.458)	241 (90.6)	0.189	1.568 (0.801-3.071)	202 (75.9)	0.011	1.857 (1.154-2.991)
Race:									
**Malay**			1			1			1
**Non-Malay**	182 (92.4)	0.168	0.499 (0.186-1.339)	171 (86.8)	0.123	0.581 (0.292-1.157)	140 (71.1)	0.469	1.193 (0.740-1.923)
Marital Status:									
**Single/Divorced**			1			1			1
**Married**	189 (96.4)	0.875	1.093 (0.361-3.315)	171 (87.2)	0.732	0.864 (0.376-1.989)	151 (77.0)	0.132	1.555 (0.876-2.763)
Education:									
**Primary/Secondary School**			1			1			1
**College/University**	278 (95.9)	0.028	2.741 (1.116-6.730)	258 (89.0)	0.855	0.933 (0.443-1.966)	216 (74.5)	0.043	1.670 (1.017-2.742)
Occupation:									
**Medical**			1			1			1
**Non-medical**	248 (95.0)	0.592	1.238 (0.567-2.707)	234 (89.7)	0.607	0.851 (0.459-1.576)	186 (71.3)	0.776	0.941 (0.619-1.431)

### Association between Preference of face masks and KAP Scores

None of the choices of preferred face masks was found to be statistically significant in association with the knowledge of the participants of this study. It was noted that the preference for N95/KN95 (p=0.046) was significantly associated with a satisfactory attitude, while the preference for cloth face masks (p=0.003) was associated with a satisfactory practice. (Table 6).

**Table 6:** Association between satisfactory knowledge, attitude, and practice on the use of face masks and the preferences of mass

Preferences of Face Masks	Knowledge			Attitude			Practice		
	n (%)	p-value	OR (95%CI)	n (%)	p-value	OR (95%CI)	n (%)	p-value	OR (95%CI)
**Surgical face mask:**									
**No**			1			1			1
**Yes**	308 (95.1)	0.216	2.001 (0.667-6.003)	291 (89.8)	0.637	1.230 (0.521-2.902)	233 (71.9)	0.573	0.828 (0.428-1.598)
**Cloth face mask:**									
**No**			1			1			1
**Yes**	79 (95.2)	0.314	1.909 (0.543-6.711)	75 (90.4)	0.657	1.220 (0.508-2.927)	47 (56.6)	0.003	0.425 (0.243-0.744)
**N95/KN95:**									
**No**			1			1			1
**Yes**	77 (91.7)	0.606	0.767 (0.280-2.103)	69 (82.1)	0.046	0.473 (0.227-0.988)	63 (75.0)	0.436	1.265 (0.700-2.288)
**Sponge face mask:**									
**No**			1			1			1
**Yes**	8 (80.0)	0.061	0.189 (0.033-1.080)	9 (90.0)	0.839	1.252(0.144-10.902)	4 (40.0)	0.121	0.346 (0.090-1.323)
**Other:**									
**No**			1			1			1
**Yes**	4 (100.0)	0.999	101763254.686 (0.000)	3 (75.0)	0.330	0.316 (0.031-3.219)	4 (100.0)	0.999	647765110.711 (0.000)

### Correlation between KAP Scores

The mean scores (SD) for knowledge, attitude, and practice on the use of face masks are 10.70 (1.3), 25.13 (3.4), and 10.77 (2.34), respectively. A weak correlation was noted between all the variables with a correlation coefficient, as summarised in Table 7.

**Table 7:** Results of Spearman's correlation coefficient analysis

Variables	Correlation Coefficient	p-value
Knowledge vs attitude	0.170	<0.001
Knowledge vs practice	0.271	<0.001
Attitude vs practice	0.202	<0.001

## Discussion

Our study found satisfactory knowledge, attitude and practice of face mask usage among the sample population. Additionally, better knowledge is significantly associated with college or university education, while better practices are associated with the female gender and college or university education. We have observed a significant but weak correlation between knowledge, attitude and practices through this study.

In this study, surgical face masks are the preferred choice, possibly due to their affordable cost and easy availability. In Malaysia, the ceiling price of RM 0.70 for a three-ply surgical face mask is comparably cheaper than RM6 for an N95 respirator.^25^ Cloth masks have different prices depending on the retailer but are more expensive than surgical face masks. Due to their reusability, cloth face masks are one of the top choices of face masks amid rising concerns about the environment and finances. More options of cloth face masks are available in the market. In Malaysia, guidelines and recommendations are made clear with infographics on making non-medical cloth face masks requiring a minimum of three layers with their specific properties.^26^,^27^ Although cloth face masks are proven to have poor efficacy compared to surgical face masks, their efficacy can be improved with the right material, layering, proper fitting, and regular wash.^26^

Practice when using reusable face masks, which include respirators and cloth face masks, was found to be satisfactory in our study. This could be due to adequate public health messaging regarding reusable face masks, especially cloth face masks, as they were being encouraged as an alternative to single-use surgical face masks amid the PPE shortage crisis in the hospital when COVID-19 cases were skyrocketing.^28^ Thus, learning how to reuse reusable face masks was easy as it was disseminated widely.^29^ Furthermore, it was encouraged by multiple environmental organisations choose reusable face masks because they are more environmentally friendly.^30^

Our finding further supported that the preference for cloth face masks was associated with a satisfactory practice score. A satisfactory attitude was noted with the preference for N95/KN95 masks. As N95/KN95 face masks offer better protection against COVID-19,^5^,^6^ albeit at a higher cost, their users would have more positive attitudes towards using them, as they are willing to purchase them at a higher cost.

The majority of the participants achieved satisfactory knowledge scores regarding face masks. A WHO publication regarding WHO's actions in countries concluded that the Malaysian authorities carried out effective risk communication and community engagement regarding COVID-19 by establishing and promoting trusted information sources early on in the pandemic through daily press conferences, social media, and mass text messages.^31^ This is reflected in the rising awareness among the community, as seen in two previous studies by Azlan et al.^15^ and Alam et al.^17^, which demonstrated an acceptable level of knowledge after the onset of COVID-19. With the majority of participants having satisfactory knowledge, we also noted that the community puts knowledge into practice, with a majority (71.7%) having satisfactory practices such as always ensuring a good fit of mask and washing hands before putting on or taking off the face mask, in line with CDC and WHO recommendations.^9^,^10^ Nearly everyone reported always wearing a face mask in public venues, which contrasts with the finding by Azlan et al.^15^ a week before the Movement Control Order (MCO) in Malaysia began, where only 51.2% of participants reported wearing a face mask when going out in public. Movement Control Order, commonly called MCO, was implemented by the federal government of Malaysia in response to the pandemic involving national quarantine and cordon sanitaire measures done serially between 18 March 2020 till 1 November 2021.

Since the emergence of COVID-19, the community has been more particular about using face masks in public and healthcare facilities. The perceived health condition among the community has a curvilinear relationship with mental health disorders (insomnia, depression, anxiety, and distress), as shown by Dai et al.^32^ Perhaps the fear of contracting the COVID-19 infection has played a part in establishing good face mask practices in combination with other precautions like social distancing and using hand sanitisers.

Most participants had a satisfactory attitude towards using face masks, although it is a relatively new habit introduced to the general public. Initially, the WHO advised masks to be worn only by medical workers or people who showed symptoms of coughing and sneezing.^9^ However, as cases continued to surge worldwide, experts decided that there was a potential benefit of face masks in preventing transmission of COVID-19. They recommended non-medical face coverings for the public.^9^ Many found it difficult at first to adapt to the habit because they would have to find and purchase the masks, put them on and dispose of them properly. Moreover, they are uncomfortable to be worn.^33^ The Malaysian government mandated face mask use in public in August 2020. Those who fail to comply can be fined up to RM1,000 and 6 months of imprisonment under the Prevention and Control of Infectious Diseases Act 1988.^34^. Therefore, this could be one of the contributing factors to a satisfactory attitude in the community. In terms of disposing of face masks, 8.4% of the community was found to be careless. Incorrect disposal of face masks can be potentially hazardous for those handling the waste. Since the emergence of COVID-19, it was found by Sangkham^35^ that the amount of used face masks and medical waste produced has increased along with the rise of confirmed cases. The increasing use of face masks was found to cause microplastic pollution on the aquatic biota described by an article by Aragraw^36^ released in July 2020. Those working in the waste management industry are also exposed to fatal pathogens with improper face mask disposal.^37^

There was no significant outcome difference between participants, as demographic variables were not statistically significant to most KAP scores. This could indicate that the community has good risk awareness and has adapted well to this new norm. More females have achieved satisfactory practice when using face masks, similar to previous studies by Guzek^38^ and one during the severe acute respiratory syndrome (SARS) pandemic in 2003 by Tang et al.^39^ A survey of nearly 2,500 people found that men considered wearing a face mask “shameful, not cool, a sign of weakness, and a stigma” more than women.^40^

Respondents working in the medical field did not have a significant difference in using face masks compared to those with non-medical-related occupations. This could be attributed to healthcare workers not having satisfactory practice in the first place, as demonstrated by a study by Tadesse et al.^14^ Otherwise. It could also suggest that the current public health messaging around face masks has been effective, and the community has learned to practice using them properly. College or university education participants were significantly associated with achieving satisfactory knowledge and practice scores. As stated by Raghupathi et al.^41^ and Hahn et al.,^42^ individuals with higher education levels have a better understanding of their health, ultimately leading to better health and a longer life expectancy. Educated individuals are also more likely to seek preventative care and engage in non-harmful health behaviours.^43^

Thus, focusing on improving education would aid in improving health awareness. In addition, public health information regarding face masks could be directed towards those with lower education, such as in the form of pictorials, to ensure better understanding and practice among them.

This study found a weak correlation between the KAP scores. This contrasts with one of the first studies by Zhong et al.^44^ on KAP regarding COVID-19, which found that higher knowledge scores were significantly associated with a lower likelihood of negative attitudes and potentially dangerous practices toward the pandemic. This, however, is similar to the findings in another by Azlan et al.^15^, which demonstrated weakly that higher knowledge scores were not necessarily correlated to good practice. This indicates that the public health messaging needs to not only stress disseminating accurate knowledge regarding the use of face masks but also emphasise good practice alongside fair law enforcement, good accessibility, and affordability of face masks. This will ensure proper and efficient public compliance towards preventive measures against COVID-19 or any succeeding pandemics in the future.

The limitations of our study were as follows: (1) The applied opportunistic sampling method may have led to selection bias. This may lead to inaccurate measures of social demographics as certain demographic characteristics may not be represented well. (2) This study excluded the illiterate population as it involved a self-administered questionnaire, which is significant as they are an important target population for interventions surrounding KAP of face mask use. (3) The association between KAP and preference for face masks may not reflect reality due to the structure of our questionnaire.

## Conclusion

There is satisfactory knowledge, attitude, and practice of face mask usage among Malaysia's Seremban 2 adult population with a weak correlation between knowledge, attitude and practices. This suggests that the public health messaging strategies have been effective in educating the public regarding correct face mask usage to decrease the transmission of COVID-19. However, this does not necessarily translate to good attitudes and practices of face mask use, as a small percentage of participants still demonstrated careless and incorrect disposal of face masks.

The results of this study may also help devise further plans for community health education with more emphasis on applying knowledge to translate to good attitudes and practices when using face masks.
